# SIRT5‐mediated HOXA5 desuccinylation inhibits ferroptosis to alleviate sepsis induced‐lung injury

**DOI:** 10.1002/kjm2.12921

**Published:** 2024-12-23

**Authors:** Lei Wang, Heng Fan, Min Sun, Ji‐Hui Ye

**Affiliations:** ^1^ Department of Critical Care Medicine The First Affiliated Hospital of Ningbo University Ningbo Zhejiang China

**Keywords:** desuccinylation, ferroptosis, HOXA5, sepsis‐induced lung injury, SIRT5

## Abstract

Acute lung injury (ALI) is a common and severe complication of sepsis with a high mortality rate. Ferroptosis, an iron‐dependent form of cell death, contributes to lung injury. Homeobox A5 (HOXA5) is involved in the regulation of septic acute kidney damage; however, its function on ferroptosis in septic ALI remains unclear. An in vitro model of septic lung injury was established in the pulmonary epithelial cell line (MLE‐12) via lipopolysaccharide (LPS) stimulation. Cell viability, ferrous iron (Fe^2+^) level, and cellular lipid reactive oxygen species (ROS) were determined with Cell Counting Kit‐8 assay, iron assay kit, and BODIPY™ 665/676 molecular probe, respectively. HOXA5, ferroptosis suppressor protein 1 (FSP1), sirtuin 5 (SIRT5), and glutathione peroxidase 4 (GPX4) expressions were measured using western blotting and Real‐Time Quantitative Polymerase Chain Reaction (RT‐qPCR. Chromatin immunoprecipitation and luciferase reporter assays were performed to validate HOXA5 binding to the FSP1/GPX4 promoter, and regulation of SIRT5 on HOXA5 desuccinylation was confirmed through co‐immunoprecipitation. LPS stimulation induced ferroptosis (reduced cell viability, elevated Fe^2+^ and lipid ROS levels, and decreased GPX4 levels) and downregulated FSP1 and HOXA5 protein levels. *HOXA5* overexpression neutralized LPS‐induced ferroptosis. Moreover, LPS exposure inhibited HOXA5 binding to the *FSP1* promoter, which was counteracted via *HOXA5* overexpression. Furthermore, SIRT5 overexpression suppressed LPS‐induced ferroptosis. In LPS‐challenged MLE‐12 cells, SIRT5‐mediated HOXA5 desuccinylation was reduced. *HOXA5* depletion neutralized the suppressive role of *SIRT5* overexpression in LPS‐induced ferroptosis. SIRT5‐mediated HOXA5 desuccinylation inhibited LPS‐induced ferroptosis by upregulating FSP1, which may offer a prospective therapeutic strategy for septic lung injury.

**Abbreviations**
ARDS: acute respiratory distress syndrome
ALI: acute lung injury
CCK‐8: Cell Counting Kit‐8
ChIP: chromatin immunoprecipitation
Co‐IP: co‐immunoprecipitation
Fer‐1: ferrostatin‐1
FSP1: ferroptosis suppressor protein 1
GPX4: glutathione peroxidase 4
HOXA5: homeobox A5
LPS: lipopolysaccharide
NAM: nicotinamide
NRF2: nuclear factor E2‐related factor 2
ROS: reactive oxygen species
SIRT5: sirtuin 5

## 1 INTRODUCTION

Sepsis, defined as a life‐threatening organ dysfunction owing to a dysregulated host response to infection,^1^ is regarded as a major cause of mortality globally.^2^ Sepsis predisposes patients to acute lung injury (ALI), which can progress to acute respiratory distress syndrome (ARDS).^3^ Recently, symptomatic therapies, including mechanical ventilation and fluid management, have become primary treatments for ALI/ARDS.^4^ However, the majority of patients with ALI/ARDS have a poor prognosis because of the lack of efficient therapeutic approaches.^5^ Hence, there is an urgent need to further investigate the pathogenesis of septic lung injury to develop effective interventions.

Ferroptosis is an iron‐dependent form of regulated cell death characterized by lipid peroxidation.^6^ Recent research has revealed the critical role of ferroptosis in the pathogenesis of septic ALI.^7^ AU‐rich element‐binding factor 1 protects against ferroptosis to relieve septic ALI by modulating activation transcription factor 3 and nuclear factor E2‐related factor 2 (NRF2)^8^; Yes‐associated protein 1 mitigates septic ALI by suppressing ferritinophagy‐mediated ferroptosis,^9^ indicating that the inhibition of ferroptosis relieves sepsis‐induced ALI. Ferroptosis suppressor protein 1 (FSP1), also known as apoptosis‐inducing factor mitochondria‐associated 2, is one of the main regulatory molecules involved in ferroptosis.^10^ FSP1 has recently been demonstrated to be a glutathione‐independent ferroptosis suppressor that promotes ferroptosis resistance by catalyzing CoQ10 regeneration using nicotinamide adenine dinucleotide phosphate hydrogen (NADPH).^11^ The upregulation of FSP1 by andrographolide can inhibit ferroptosis to alleviate acute septic kidney injury.^12^ Therefore, elevated FSP1 levels may inhibit ferroptosis and relieve sepsis‐induced lung injury.

Aberrant expression of the homeobox A5 (HOXA5) transcription factor, a member of the A cluster of HOX genes, contributes to the anomalies and dysfunction of many organs.^13^ Upon abdominal sepsis challenge or lipopolysaccharide (LPS) stimulation, proteasome‐mediated HOXA5 turnover is involved in the regulation of septic acute kidney injury.^14^ Using JASPAR (https://jaspar.elixir.no/) prediction, we found that HOXA5 binds to the *FSP1* promoter. Therefore, HOXA5 may regulate FSP1‐mediated ferroptosis and participate in sepsis‐induced lung injury.

Lysine succinylation is a newly identified post‐translational modification in which a lysine residue is modified with a succinyl group.^15^ Sirtuin 5 (SIRT5), an NAD+‐dependent protein lysine deacylase, primarily catalyzes lysine demalonylation, desuccinylation, and deglutarylation.^16^ SIRT5‐mediated desuccinylation of mitochondrial malic enzyme 2 promotes disease progression in colorectal cancer.^17^ SIRT5 desuccinylates and activates pyruvate kinase M2, which protects against colitis induced by dextran sulfate sodium in mice.^18^ Succinylation has been shown to increase sepsis‐induced liver injury.^19^ GEO database (https://www.ncbi.nlm.nih.gov/gds/) analysis shows that SIRT5 expression is decreased in sepsis‐induced lung injury. Additionally, HOXA5 succinylation is predicted, suggesting that K196/212 on HOXA5 may undergo succinylation. BioGRID4.4 (https://thebiogrid.org/) predicts an interaction between SIRT5 and HOXA5. Accordingly, we hypothesized that SIRT5 regulates HOXA5 desuccinylation in sepsis‐induced lung injury.

This study aimed to explore the role and potential mechanism of HOXA5 desuccinylation in the regulation of ferroptosis in sepsis‐induced lung injury. We demonstrated that LPS‐induced SIRT5 downregulation led to a reduction in HOXA5 desuccinylation, which inhibited *FSP1* transcriptional activation, thereby promoting ferroptosis and contributing to septic lung injury. This study provides an innovative strategy for treating septic lung injury.

## 2 MATERIALS AND METHODS

### 2.1 Cell culture

Human embryonic kidney (HEK293T) and mouse lung epithelial (MLE‐12) cells were purchased from the ATCC (Manassas, VA, USA). Cells were cultured in DMEM (Gibco, Grand Island, NY, USA) containing fetal bovine serum (10%) and 100 U/mL of streptomycin/penicillin at 37°C with 5% CO_2_.

### 2.2 Cell transfection and treatment

Overexpression vectors of *HOXA5* (OE‐*HOXA5*) and *SIRT5* (OE‐*SIRT5*) and shRNA targeting *HOXA5* (sh‐*HOXA5*) were supplied by Gene Chem (Shanghai, China). MLE‐12 cells were seeded in 24‐well plates. Referring to the instructions, MLE‐12 cells were transfected with OE‐*HOXA5*, OE‐*SIRT5*, sh‐*HOXA5*, or negative controls for 48 h.

Additionally, hemagglutinin (HA)‐labeled WT‐SIRT5 and its mutant SIRT5‐H158Y were designed by Fenghbio (Changsha, China), and Flag‐tagged SIRT5 and HOXA5 were prepared. HEK‐293T and MLE‐12 cells were transfected with the aforementioned plasmids for 24 h.

Following transfection, cells were treated with LPS (1 mg/mL, Sigma, St Louis, MO, USA) for 24 h to establish an *in vitro* lung injury model. In addition, the ferroptosis inhibitor ferrostatin‐1 (Fer‐1, 2 μM) was administered to cells for 24 h. Cells were also treated with succinyl‐CoA (0, 0.5, or 1 mM; Sigma) or pan‐sirtuin inhibitor nicotinamide (NAM, 5 mM, Sigma).

### 2.3 Cell viability determination

Cell Counting Kit‐8 (CCK‐8; Dojindo, Tokyo, Japan) was utilized to assess cell viability in accordance with the manufacturer's instructions. MLE‐12 cells (5 × 10^3^ cells/well) were seeded in a 96‐well plate and incubated for 24 h, followed by treatment. Next, CCK‐8 solution (10 μL) was added to each well, followed by incubation at 37°C for an additional 3 h. A microplate spectrophotometer was used to measure the optical density at 450 nm.

### 2.4 Western blotting

Lysis buffer was used to extract proteins from the cells. A Bicin‐choninic acid （BCA） protein assay kit (Pierce Chemical, Rockford, IL, USA) was employed to quantify protein concentrations. Protein samples from each group were separated using dodecyl sulfate, sodium salt (SDS)‐Polyacrylamide gel electrophoresis (PAGE) gels and then transferred onto Polyvinylidene Fluoride (PVDF) membranes. The membranes were blocked at room temperature for 1 h with 5% nonfat milk. Membranes were incubated with primary antibodies against HOXA5 (Invitrogen, Carlsbad, CA, USA), SIRT5 (Abcam, Cambridge, MA, USA), FSP1 (Sigma), glutathione peroxidase 4 (GPX4) (Abcam), and β‐actin (Abcam) at 4°C overnight. Subsequently, membranes were incubated with secondary antibodies (Abcam) for 1 h. An Image Quant LAS 4000 mini (GE Healthcare Bio‐Sciences AB, Uppsala, Sweden) was used to detect protein bands with an ECL kit (Beyotime, Jiangsu, China). ImageJ software was employed to evaluate the band intensities.

### 2.5 Real‐Time Quantitative Polymerase Chain Reaction （RT‐qPCR）

TRIzol reagent (Invitrogen) was used to extract total RNA from the cells. SuperScript III Reverse Transcriptase (Invitrogen) was utilized to reverse transcribe the RNA into cDNA. RT‐PCR was performed on an ABI PRISM 7900 Sequence Detection System (Applied Biosystems, Foster City, CA, USA) using the SYBR Green Master Mix (Applied Biosystems). The 2^−ΔΔCt^ method was employed to calculate the relative expressions of *SIRT5* (NM_178848.3) and *HOXA5* (NM_010453.6), with *glyceraldehyde‐3‐phosphatedehydrogenase (GAPDH)* (NM_001289726.2) acting as the internal control. Primers used are listed in Table 1.

**TABLE 1:** Primer sequences used in RT‐qPCR.

Gene	Forward primer (5′–3′)	Reverse primer (5′–3′)
*SIRT5*	GTCATCACCCAGAACATCGA	ACGTGAGGTCGCAGCAAGCC
*HOXA5*	CCCAGATCTACCCCTGGATG	CAGGGTCTGGTAGCGAGTGT
*GAPDH*	AAAGGGCATCCTGGGCTACA	CAGTGTTGGGGGCTGAGTTG

### 2.6 Detection of Fe^2+^ level

An iron assay kit (Sigma) was used to detect ferrous iron (Fe^2+^) levels according to the manufacturer's protocol. MLE‐12 cells were lysed to obtain the supernatants. Next, supernatant (100 μL) was mixed with iron buffer (5 μL) and incubated for 0.5 h at 37°C. The optical density was measured at 593 nm.

### 2.7 Lipid reactive oxygen species measurement

BODIPY™ 665/676 molecular probe (Invitrogen) was utilized to determine cellular lipid reactive oxygen species (ROS). After the indicated treatments, cells were incubated for 1 h with a culture medium (1 mL) containing BODIPY™ 665/676 reagent (10 μM). Next, the cells were resuspended in PBS (1 mL). Flow cytometry was used to analyze lipid ROS levels in cells. Cytoflex (Beckman Coulter, Brea, CA, USA) was employed to assess the oxidized BODIPY™ 665/676 probe, and CytExpert 2.4 software was used to analyze the data.

### 2.8 Chromatin immunoprecipitation

A chromatin immunoprecipitation (ChIP) assay kit (Millipore, Billerica, MA, USA) was used according to the manufacturer's guidelines. Cells were incubated in a 1% formaldehyde solution at 37°C for 20 min for DNA and protein crosslinking. After sonication, chromatin was immunoprecipitated overnight with anti‐IgG (Abcam) or anti‐HOXA5 (Santa Cruz Biotechnology, Santa Cruz, CA, USA). RT‐qPCR was performed to amplify the purified DNA fragments using specific primers for the promoter region of *FSP1* or *GPX4* containing HOXA5 binding sites (BS1 and BS2).

### 2.9 Dual‐luciferase reporter assay

Two wild‐type (WT) binding sites (BS1 and BS2) of HOXA5 on the *FSP1* promoter, mutant BS1 sequence (MUT1), mutant BS2 sequence (MUT2), or mutant BS1 and BS2 sequences (MUT1 and MUT2) were cloned into pGL3 luciferase reporter plasmids (Promega, Madison, WI, USA). A Dual‐Luciferase Reporter Assay Kit (Promega) was utilized to perform the luciferase reporter assay according to the manufacturer's recommendations. Briefly, cells (1 × 10^4^ cells per well) were seeded in a 96‐well plate, followed by co‐transfection with 100 ng of the luciferase reporter plasmids and OE‐*HOXA5* or OE‐NC using Lipofectamine 3000 reagent for 48 h. Luciferase activity was evaluated after the cells were collected.

### 2.10 Co‐immunoprecipitation

The role of SIRT5 in regulating HOXA5 succinylation was validated through co‐immunoprecipitation (Co‐IP) in MLE‐12 and HEK293T cells. Radio immunoprecipitation assay (RIPA) buffer (Beyotime) containing a protease inhibitor was used to lyse the cells for 0.5 h. Subsequently, the supernatants were incubated at 4°C overnight with anti‐Flag (Millipore), anti‐SIRT5 (Abcam), anti‐HOXA5 (Santa Cruz), or anti‐lgG (Abcam). Protein A agarose beads (Thermo, Waltham, MA, USA) were then added, followed by a 2‐h incubation at 4°C. The complexes were centrifugated at 3000 rpm for 3 min at 4°C, and the supernatants were discarded. After rinsing the agarose beads with lysis buffer, western blotting was performed to detect the precipitated proteins.

### 2.11 Statistical analysis

Statistical analysis was conducted using SPSS 26.0 software. Data are displayed as means ± SD. Student's *t*‐test was used for comparisons between the two groups. One‐way analysis of variance followed by Tukey's post hoc test was applied for comparisons among multiple groups. Statistical significance was set at *p* < 0.05.

## 3 RESULTS

### 3.1 Overexpressing *HOXA5* inhibited ferroptosis in an *in vitro* model of LPS‐induced septic lung damage

**FIGURE 1:**
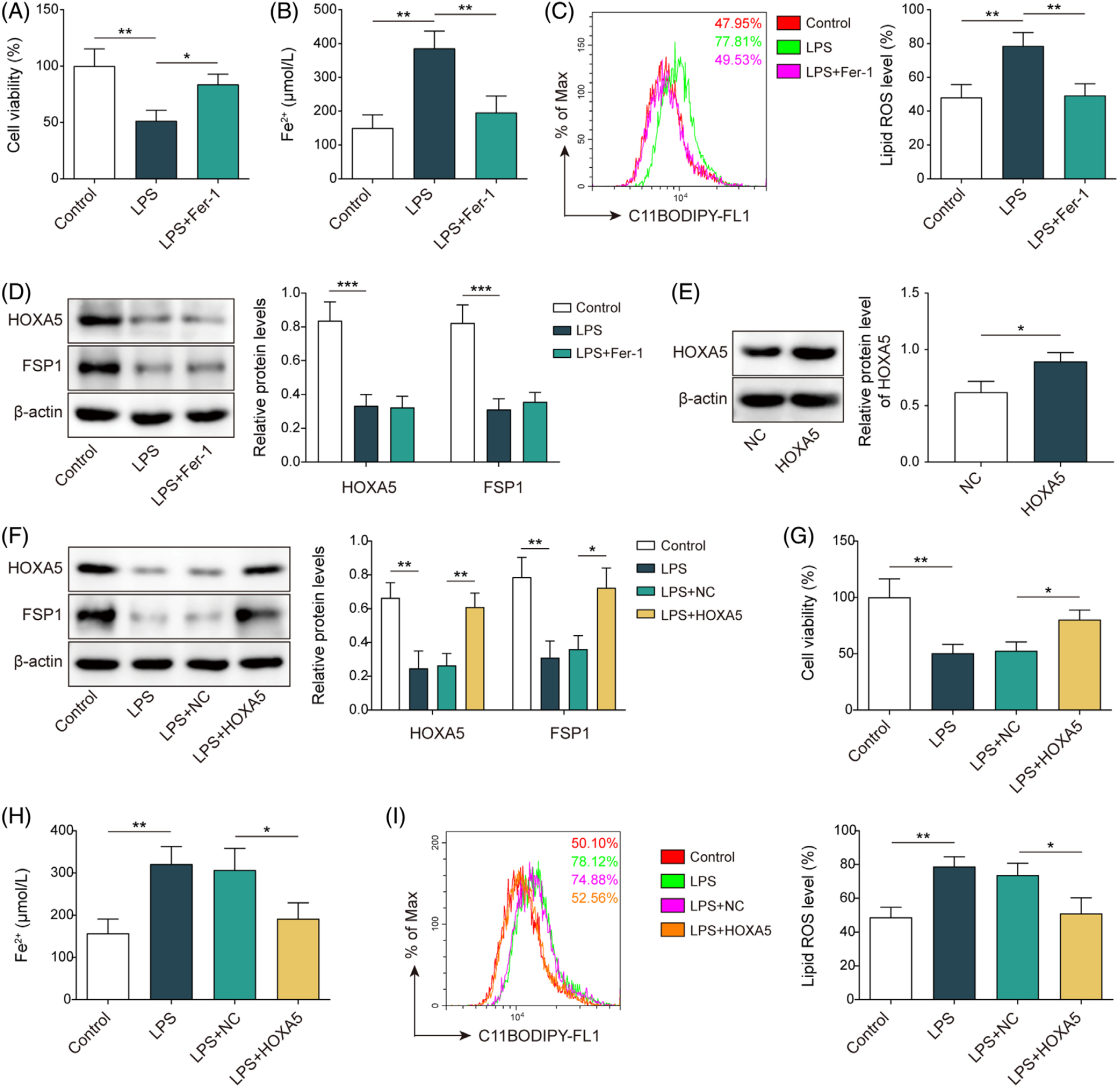
*HOXA5* overexpression suppresses ferroptosis in LPS‐induced septic lung injury *in vitro*. (A–D) MLE‐12 cells were subjected to LPS stimulation, followed by treatment with Fer‐1. CCK‐8 detected cell viability (A). An iron assay kit measured Fe^2+^ levels (B). Lipid ROS determined with BODIPY™ 665/676 dye (C). Western blotting detected HOXA5 and FSP1 protein levels (D). (E) *HOXA5* overexpression vector (OE‐*HOXA5*) were transfected into MLE‐12 cells, and western blotting was employed to test HOXA5 protein level. (F–I) Transfected cells were stimulated by LPS. Western blotting analyzed HOXA5 and FSP1 protein levels (F). CCK‐8 kit determined cell viability (G). Fe^2+^ level was measured using an iron assay kit (H). Lipid ROS determined with BODIPY™ 665/676 dye (I). *p < 0.05, **p < 0.01, ***p < 0.001. *n* = 3. Data are shown as mean ± SD. CCK‐8, Cell Counting Kit‐8; FSP1, ferroptosis suppressor protein 1; HOXA5, homeobox A5; LPS, lipopolysaccharide; MLE‐12, mouse lung epithelial cells; ROS, reactive oxygen species.

To verify the alteration of ferroptosis in septic lung injury, MLE‐12 cells were stimulated with LPS to establish a septic lung injury cell model before treatment with the ferroptosis inhibitor, Fer‐1. Cell viability was reduced in LPS‐induced MLE‐12 cells compared to that in the control group; however, Fer‐1 treatment increased cell viability (Figure 1A). LPS treatment increased the Fe^2+^ content, which declined after the addition of Fer‐1 (Figure 1B). Furthermore, LPS led to an abundant accumulation of lipid ROS in MLE‐12 cells, whereas Fer‐1 counteracted this effect (Figure 1C). LPS stimulation decreased the protein levels of GPX4, a key negative regulator of ferroptosis, which were elevated via Fer‐1 treatment (Figure S1A). These data indicate the promotion of ferroptosis in LPS‐induced lung injury. HOXA5 and FSP1 protein levels were downregulated by LPS but were not altered upon treatment with Fer‐1 (Figure 1D). HOXA5 expression was elevated in cells transfected with OE‐*HOXA5* compared to that in the OE‐NC group (Figure 1E), indicating the effective overexpression of HOXA5. To study the function of HOXA5 in septic lung injury, the cells transfected with OE‐*HOXA5* were treated with LPS. The decrease in LPS‐induced HOXA5 and FSP1 were reversed when *HOXA5* was overexpressed (Figure 1F). *HOXA5* overexpression reversed the LPS‐induced reduction in cell viability (Figure 1G). Furthermore, elevated levels of Fe^2+^ and lipid ROS in LPS‐treated cells were neutralized by *HOXA5* overexpression (Figure 1H,I). The LPS‐induced decrease in GPX4 expression was counteracted by *HOXA5* overexpression (Figure S1B). These results indicated that *HOXA5* overexpression repressed ferroptosis in LPS‐challenged MLE‐12 cells.

### 3.2 HOXA5 bound to the *FSP1* promoter to suppress ferroptosis in LPS‐stimulated septic lung damage model *in vitro*

**FIGURE 2:**
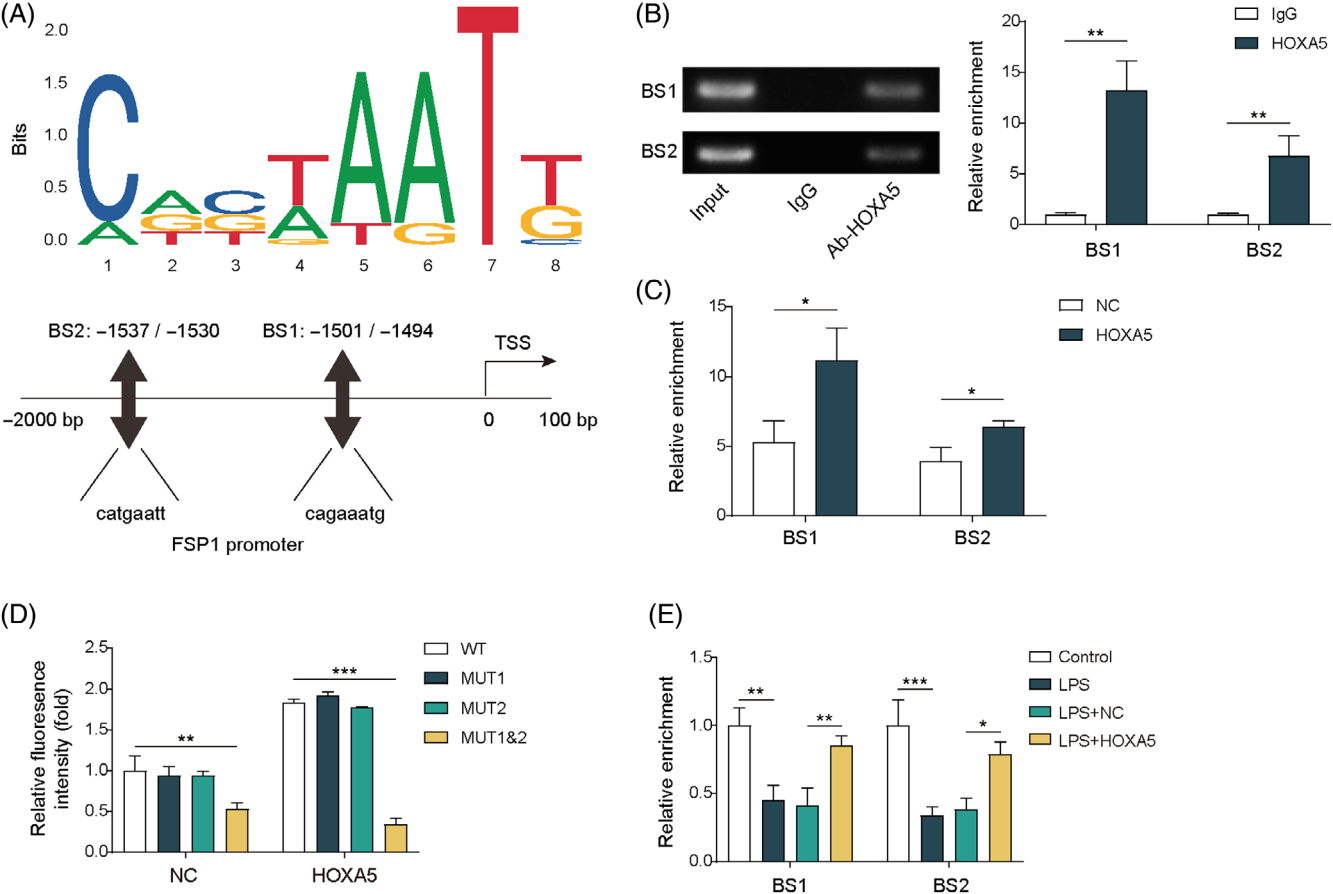
HOXA5 binds to the *FSP1* promoter region to inhibit ferroptosis in LPS‐induced septic lung injury *in vitro*. (A) HOXA5 binding sites, including BS1 (−1501/−1494, 5′‐cagaaatg‐3′) and BS2 (−1537/−1530, 5′‐catgaatt‐3′), in the *FSP1* gene promoter predicted with JASPAR database. (B) ChIP analysis validated HOXA5 binding to the promoter of *FSP1* in MLE‐12 cells. (C) ChIP assay determined HOXA5 binding to *FSP1* promoter in MLE‐12 cells with *HOXA5* overexpression. (D) MLE‐12 cells were transfected with OE‐*HOXA5*, and dual‐luciferase activity was determined through dual‐luciferase assay. (E) MLE‐12 cells transfected with or without OE‐*HOXA5* were subjected to LPS stimulation. ChIP assay was performed for measuring HOXA5 binding to *FSP1* promoter. *p < 0.05, **p < 0.01, ***p < 0.001. *n* = 3. Data are shown as mean ± SD. BS1, binding site 1; BS2, binding site 2; ChIP, chromatin immunoprecipitation; FSP1, ferroptosis suppressor protein 1; HOXA5, homeobox A5; LPS, lipopolysaccharide; MLE‐12, mouse lung epithelial cells.

We then determined whether HOXA5 modulates ferroptosis in septic lung injury by regulating FSP1 expression. Two binding sites (BS1 and BS2) of HOXA5 in the *FSP1* promoter were predicted using the JASPAR database (Figure 2A). The ChIP assay revealed that the enrichment of BS1 and BS2 was both increased in MLE‐12 cells when using anti‐HOXA5, with higher enrichment of BS1 (Figure 2B), indicating that HOXA5 bound to BS1 and BS2 of the *FSP1* promoter. Moreover, compared to the OE‐NC group, increased HOXA5 occupancy at the BS1 and BS2 positions of the *FSP1* promoter was observed in *HOXA5*‐overexpressed cells (Figure 2C). Next, a dual‐luciferase reporter assay revealed that overexpression of *HOXA5* could elevate luciferase activity in WT, MUT1, or MUT2‐transfected cells, whereas no alteration was observed in MUT1‐ and MUT2‐transfected cells after *HOXA5* overexpression (Figure 2D). LPS exposure led to reduced enrichment of BS1 or BS2 at the FSP1 promoter, whereas *HOXA5* overexpression counteracted these effects, as verified through the ChIP assay (Figure 2E). These data indicate that HOXA5 inhibits ferroptosis by binding to the *FSP1* promoter in LPS‐stimulated MLE‐12 cells. Additionally, we explored whether HOXA5 was involved in the regulation of GPX4 to affect ferroptosis in septic lung injury. Using the JASPAR database, two HOXA5 binding sites, BS1 and BS2, were predicted in the *GPX4* promoter (Figure S2A). The binding of HOXA5 to BS1 and BS2 on the *GPX4* promoter was validated using the ChIP assay, with higher enrichment of BS2 (Figure S2B); the occupancies of HOXA5 at BS1 and BS2 of the *GPX4* promoter were increased when *HOXA5* was overexpressed, while higher enrichment was observed at the BS2 position (Figure S2C). LPS treatment decreased the enrichment of BS1 and BS2 in the *GPX4* promoter, which was antagonized by *HOXA5* overexpression (Figure S2D). Collectively, HOXA5 may suppress ferroptosis by interacting with the *GPX4* promoter during septic lung injury.

### 3.3 Overexpressing *SIRT5* repressed ferroptosis in the *in vitro* LPS‐induced septic lung injury model

**FIGURE 3:**
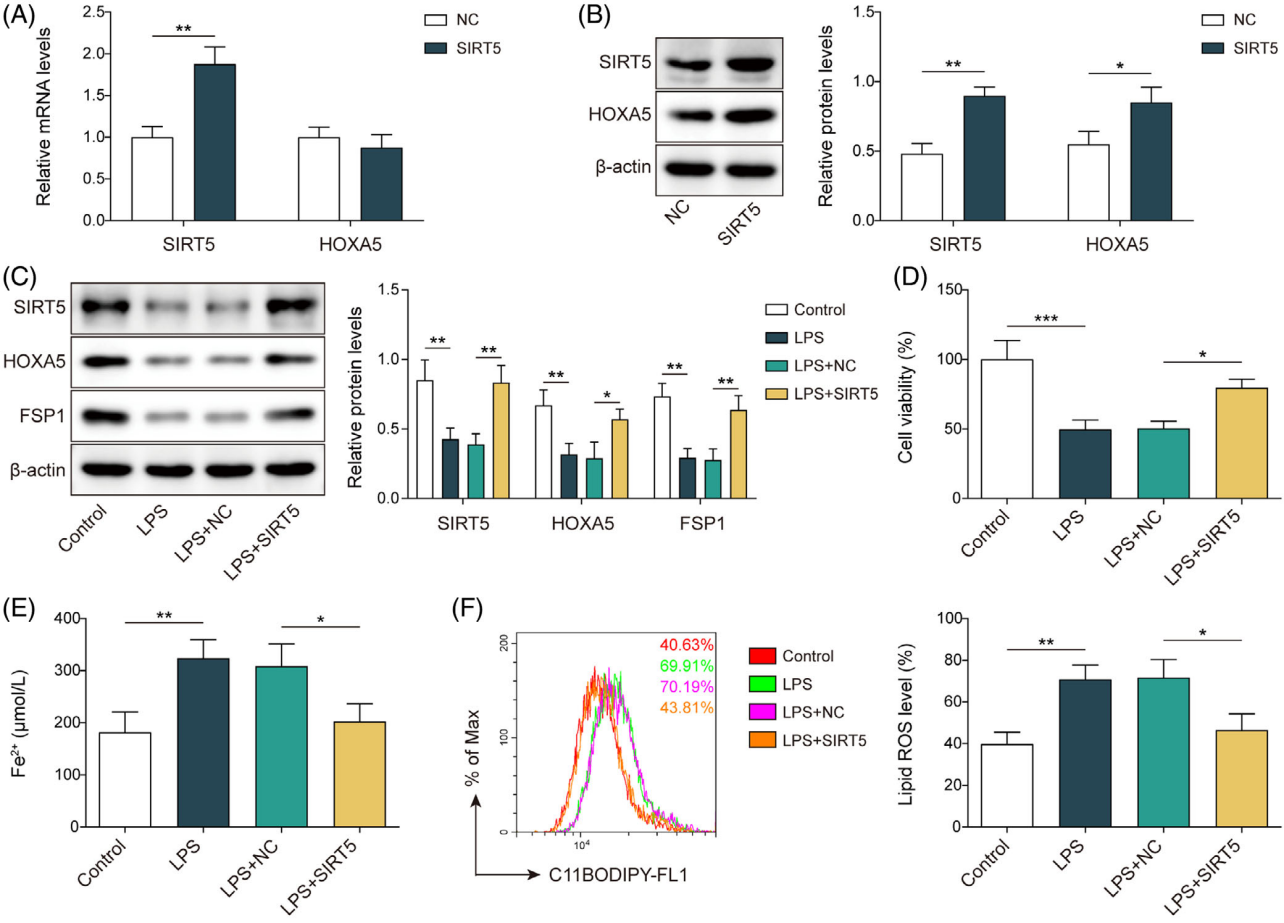
*SIRT5* overexpression suppresses ferroptosis in LPS‐induced septic lung damage in vitro. (A, B) MLE‐12 cells were transfected with *SIRT5* overexpression vector (OE‐*SIRT5*). RT‐qPCR (A) and western blotting (B) analyzed SIRT5 and HOXA5 expressions at mRNA and protein levels. (C–F) MLE‐12 cells transfected with OE‐*SIRT5* were exposed to LPS. (C) Western blotting measured the protein expressions of SIRT5, HOXA5, and FSP1. (D) CCK‐8 tested cell viability. (E) Fe^2+^ level was analyzed with an iron assay kit. (F) BODIPY™ 665/676 dye was used to detect lipid ROS level. *p < 0.05, **p < 0.01, ***p < 0.001. *n* = 3. Data are shown as mean ± SD. CCK‐8, Cell Counting Kit‐8; FSP1, ferroptosis suppressor protein 1; HOXA5, homeobox A5; LPS, lipopolysaccharide; MLE‐12, mouse lung epithelial cells; ROS, reactive oxygen species; SIRT5, sirtuin 5.

To determine the function of SIRT5 in the regulation of ferroptosis in septic lung injury, we overexpressed *SIRT5* in MLE‐12 cells. Cells transfected with OE‐*SIRT5* showed upregulation of *SIRT5* mRNA levels; however, *HOXA5* mRNA levels were not altered (Figure 3A). Notably, upregulated protein levels of both SIRT5 and HOXA5 were observed following *SIRT5* overexpression (Figure 3B). Furthermore, in LPS‐stimulated cells, the protein levels of SIRT5, HOXA5, and FSP1 decreased; however, overexpression of *SIRT5* led to the upregulation of SIRT5, HOXA5, and FSP1 (Figure 3C). In MLE‐12 cells, *SIRT5* overexpression antagonized the decrease in cell viability (Figure 3D) and elevated Fe^2+^ (Figure 3E) and lipid ROS (Figure 3F) levels induced by LPS treatment. When *SIRT5* was overexpressed, the downregulation of GPX4 elicited by LPS was reversed (Figure S1C). Collectively, these data indicate that SIRT5 inhibits ferroptosis in LPS‐stimulated MLE‐12 cells.

### 3.4 SIRT5‐mediated HOXA5 desuccinylation was reduced in the *in vitro* LPS‐induced septic lung injury model

**FIGURE 4:**
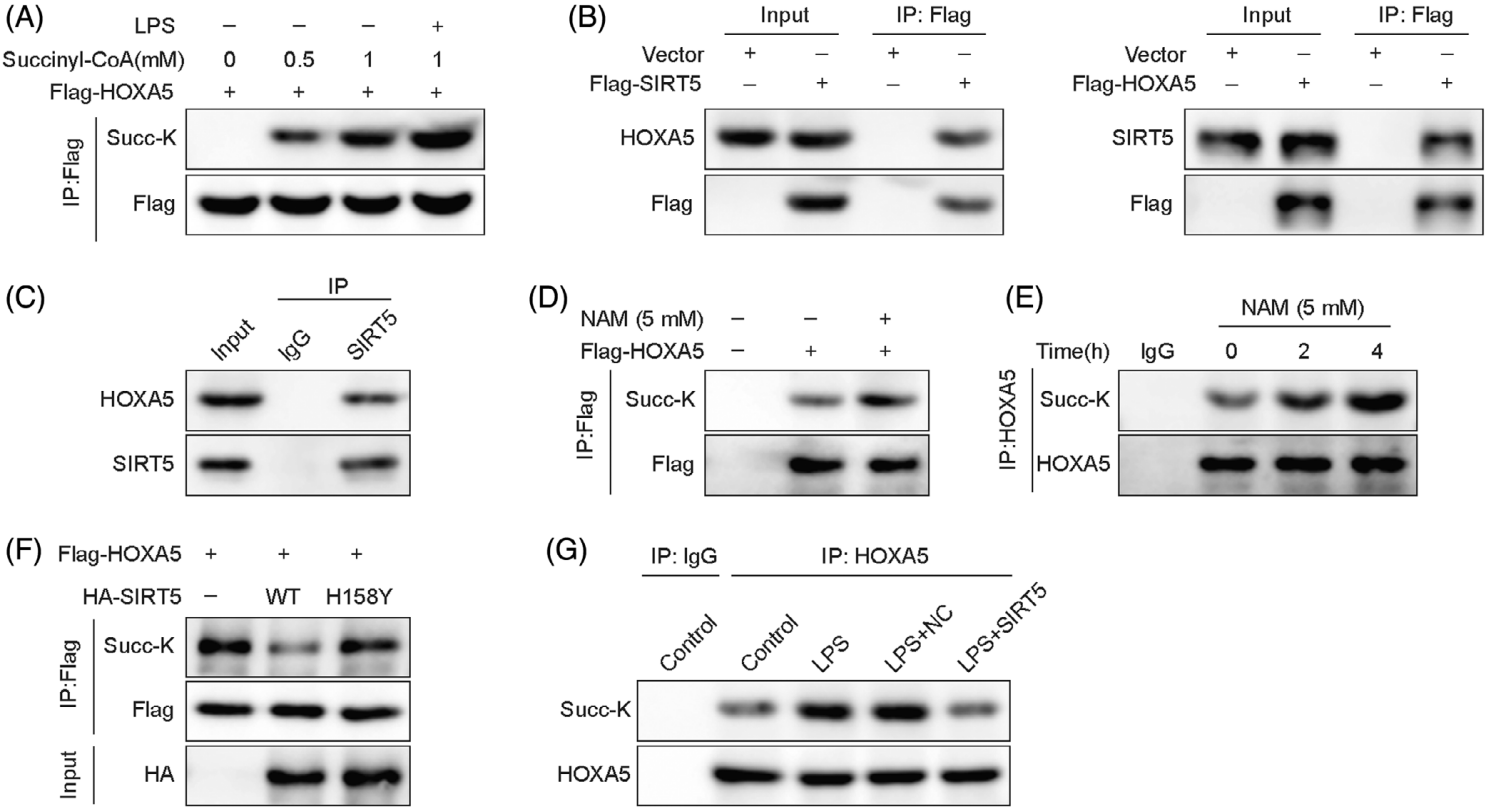
SIRT5‐mediated HOXA5 desuccinylation is decreased in LPS‐induced septic lung injury *in vitro*. (A) MLE‐12 cells were treated with succinyl‐CoA (0, 0.5, or 1 mM) or with 1 mM succinyl‐CoA and LPS. Co‐IP measured succinylation of HOXA5. (B) Flag‐tagged SIRT5 or Flag‐tagged HOXA5 was transfected into HEK293T cells, and then Co‐IP was performed to assess the interaction of SIRT5 and HOXA5. (C) In MLE‐12 cells, the combination of SIRT5 and HOXA5 was detected using Co‐IP. (D) HEK293T cells transfected with Flag‐labeled HOXA5 were treated with NAM (5 mM). HOXA5 succinylation was assessed via Co‐IP. (E) MLE‐12 cells were incubated with NAM (5 mM) for 0, 2, or 4 h. Co‐IP was then performed to evaluate HOXA5 succinylation. (F) HEK293T cells were transfected with Flag‐labeled HOXA5, HA‐labeled WT‐SIRT5, or its mutant SIRT5‐H158Y. HOXA5 succinylation was determined using Co‐IP assay. (G) MLE‐12 cells transfected with OE‐*SIRT5* were subjected to LPS stimulation. Co‐IP analysis of HOXA5 succinylation. Co‐IP, co‐immunoprecipitation; HOXA5, homeobox A5; LPS, lipopolysaccharide; MLE‐12, mouse lung epithelial cells; NAM, nicotinamide; SIRT5, sirtuin 5.

Subsequently, we validated whether SIRT5 regulates the desuccinylation of HOXA5 using a series of Co‐IP assays. The succinylation of HOXA5 increased with higher concentrations of succinyl‐CoA in MLE‐12 cells and was further elevated upon LPS stimulation (Figure 4A).

In HEK293T cells, HOXA5 was coimmunoprecipitated by Flag‐tagged SIRT5, and in turn, Flag‐tagged HOXA5 was able to precipitate SIRT5 (Figure 4B). Moreover, in MLE‐12 cells, HOXA5 was detected in the Co‐IP product using an anti‐SIRT5 antibody (Figure 4C), indicating an interaction between SIRT5 and HOXA5. Subsequently, NAM, a pan‐sirtuin inhibitor, was added into HEK293T cells transfected with Flag‐labeled HOXA5. The results showed that the upregulation of HOXA5 succinylation occurred upon NAM treatment (Figure 4D). Furthermore, the time‐dependent elevation of HOXA5 succinylation induced by NAM was further confirmed in MLE‐12 cells (Figure 4E). Additionally, a reduction in HOXA5 succinylation was induced by WT‐SIRT5 in HEK293T cells but was restored by transfection with mutant SIRT5‐H158Y in HEK293T cells (Figure 4F). Meanwhile, LPS treatment led to the upregulation of HOXA5 succinylation in MLE‐12 cells, which was reversed when SIRT5 was overexpressed (Figure 4G). Collectively, these results indicated that decreased SIRT5 was responsible for the downregulation of HOXA5 desuccinylation in LPS‐induced MLE‐12 cells.

### 3.5 *SIRT5* overexpression repressed ferroptosis via HOXA5 in LPS‐stimulated septic lung damage model *in vitro*

**FIGURE 5:**
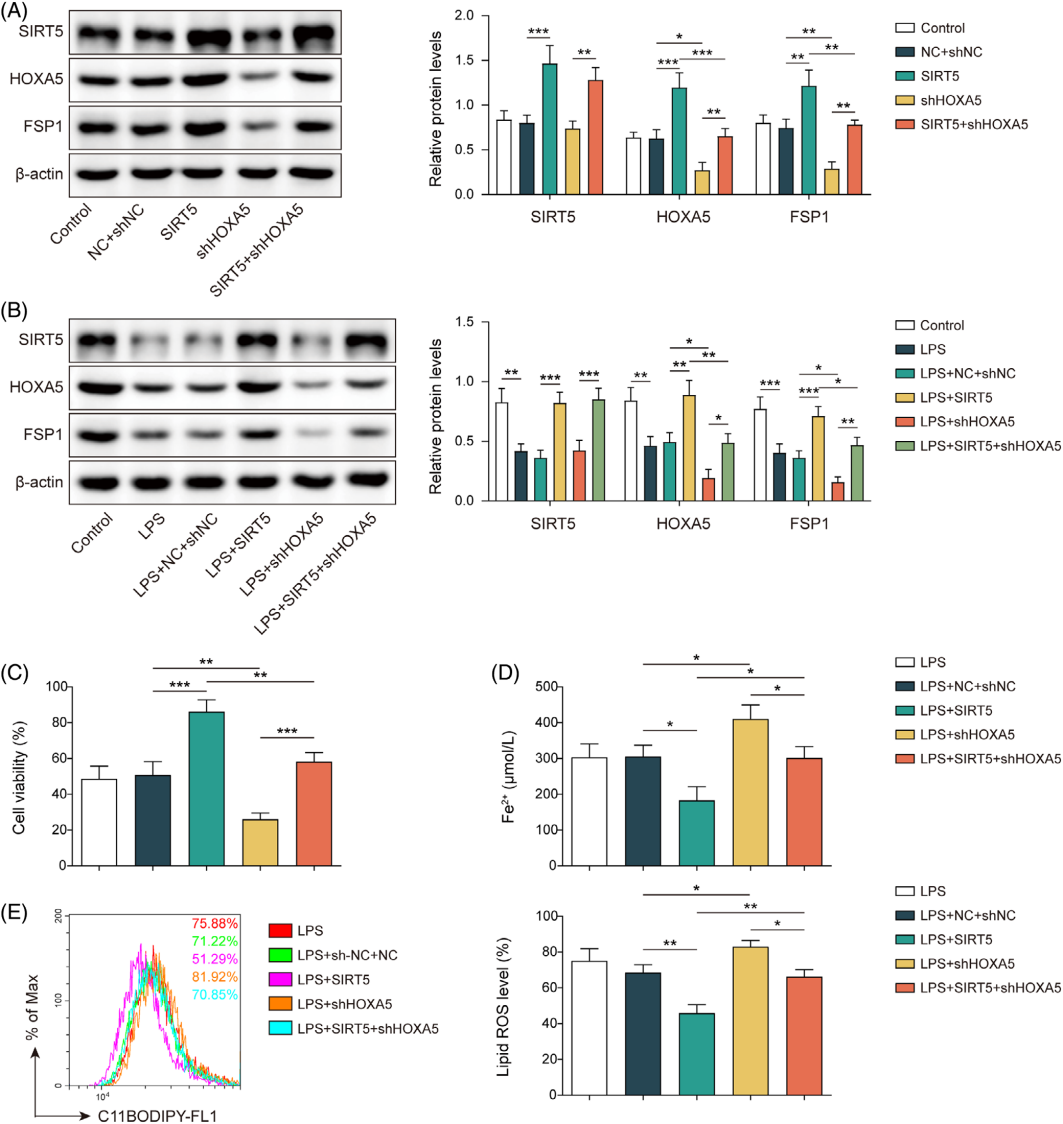
SIRT5 inhibits ferroptosis via HOXA5 in LPS‐induced septic lung damage cell model. (A) MLE‐12 cells were transfected with OE‐SIRT5 or/and sh‐HOXA5. Western blotting tested SIRT5, HOXA5, and FSP1 protein expressions. (B–E) Transfected cells were subjected to LPS stimulation. (B) Western blotting determined SIRT5, HOXA5, and FSP1 protein expressions. (C) CCK‐8 detected cell viability. (D) Fe^2+^ level was measured using an iron assay kit. (E) Lipid ROS was assessed with BODIPY™ 665/676 dye. *p < 0.05, **p < 0.01, ***p < 0.001. *n* = 3. Data are shown as mean ± SD. CCK‐8, Cell Counting Kit‐8; FSP1, ferroptosis suppressor protein 1; HOXA5, homeobox A5; LPS, lipopolysaccharide; MLE‐12, mouse lung epithelial cells; ROS, reactive oxygen species; SIRT5, sirtuin 5.

To explore whether HOXA5 is involved in SIRT5‐mediated modulation of ferroptosis, MLE‐12 cells were transfected with OE‐*SIRT5* and/or sh‐*HOXA5*. In the absence of LPS, *SIRT5* overexpression led to elevated protein levels of HOXA5 and FSP1. Meanwhile, cells transfected with sh‐*HOXA5* exhibited reductions of HOXA5 and FSP1, but no alteration of SIRT5 was observed. Moreover, the knockdown of *HOXA5* reversed the increase in HOXA5 and FSP1 levels induced by *SIRT5* overexpression (Figure 5A). Consistent results were obtained in the presence of LPS. LPS‐induced reductions in HOXA5 and FSP1 were reversed by *SIRT5* overexpression but enhanced by HOXA5 silencing. Silencing *HOXA5* counteracted the effects of *SIRT5* overexpression (Figure 5B). Decreased cell viability and elevated Fe^2+^ and lipid ROS levels induced by LPS were reversed by *SIRT5* was overexpressed and enhanced by HOXA5 knockdown. Furthermore, the effects of *SIRT5* overexpression were neutralized following HOXA5 depletion (Figure 5C–E). The downregulation of GPX4 elicited by LPS was reversed by *SIRT5* overexpression but strengthened by *HOXA5* silencing. Additionally, the knockdown of *HOXA5* reversed the effects of *SIRT5* overexpression (Figure S1D). Collectively, these findings suggest that SIRT5 suppresses ferroptosis via upregulation of HOXA5 in LPS‐stimulated MLE‐12 cells.

## 4 DISCUSSION

Sepsis‐induced ALI, characterized by widespread lung dysfunction, is associated with significant morbidity and mortality owing to limited treatment options.^20^ Ferroptosis plays an essential role in ALI.^21^ Herein, we confirmed that HOXA5 effectively suppressed ferroptosis in a cell model of LPS‐induced septic lung damage. Furthermore, SIRT5‐mediated HOXA5 desuccinylation alleviated LPS‐induced septic lung injury *in vitro* by repressing ferroptosis, which depends on the activation of *FSP1* transcription.

Reducing HOXA1 expression by UCA1 overexpression aggravates the progression of sepsis‐induced pneumonia.^22^ By targeting HOXA1, HOTAIRM1 blockade alleviates lung injury and improves the survival of mice in an in vivo model of sepsis.^23^ Dismantling the proteasome‐mediated turnover of HOXA5 contributes to the prevention of septic acute kidney injury.^14^ However, its role in sepsis‐induced lung injury remains unclear. In the present study, we observed the downregulation of HOXA5 in LPS‐challenged lung epithelial cells (MLE‐12). Importantly, in the in vitro model of LPS‐induced septic lung damage, our study validated the positive function of HOXA5 in ferroptosis suppression, as indicated by the increase in cell viability, reduction of Fe^2+^ and lipid ROS, and increase in GPX4. These findings suggest that HOXA5 ameliorates septic lung injury by suppressing ferroptosis. Activating the NRF2/FSP1 pathway with andrographolide inhibits ferroptosis to attenuate sepsis‐induced acute kidney damage.^12^ Ginsenoside Rg1 inhibits the ferroptosis of renal tubular epithelial cells to alleviate acute kidney damage induced by sepsis by elevating FSP1,^24^ as well as via the FSP1‐CoQ10‐NADPH pathway.^25^ Acetaminophen inhibits ferroptosis in the cerebral hippocampus of mice with sepsis, at least in part, through the FSP1 pathway.^26^ In the present study, the binding of HOXA5 to the *FSP1* promoter was predicted using the JASPAR software. LPS was found to reduce FSP1 in lung epithelial cells, whereas *HOXA5* overexpression upregulated FSP1. Using a dual‐luciferase reporter assay, we demonstrated that HOXA5 promoted *FSP1* transcriptional activity. LPS inhibited HOXA5 binding to the *FSP1* promoter, whereas *HOXA5* overexpression reversed this effect. Accordingly, this study proposes that HOXA5 reduces ferroptosis by binding to the *FSP1* promoter in an LPS‐induced *in vitro* septic lung injury model. In addition, overexpression of *HOXA5* reversed the repressed HOXA5 binding to the *GPX4* promoter caused by LPS. Therefore, this study also found that HOXA5 may inhibit ferroptosis via interaction with the *GPX4* promoter in septic lung injury.

Upregulation of SIRT5 expression alleviates septic acute kidney injury by enhancing AMPK phosphorylation.^27^ Additionally, SIRT5 regulates ferroptosis through NRF2/heme oxygenase‐1 signaling and participates in ischemia–reperfusion injury in ischemic stroke. Conversely, *SIRT5* knockdown prevents erastin‐induced cell ferroptosis and elevates GPX4.^28^ Aggravated hepatic pathological injury is observed in *SIRT5* knockout mice, accompanied by increased iron levels and GPX4 expression.^29^ Our study revealed that upon LPS stimulation, *SIRT5* overexpression upregulated HOXA5 and FSP1 expression in lung epithelial cells. Notably, in an LPS‐induced *in vitro* model of septic lung injury, *SIRT5* overexpression inhibited ferroptosis, as demonstrated by elevated cell viability, reduced Fe^2+^ and lipid ROS, and increased GPX4. Thus, the present study demonstrated that SIRT5 plays an essential role in ferroptosis suppression in septic lung injury.

Recently, SIRT5 has drawn broad attention because of its specific catalytic activity in significant desuccinylation.^30^ The protective function of SIRT5 in acute kidney injury induced by sepsis may be partly ascribed to its desuccinylating action on ATPase inhibitory factor 1.^31^ Inhibition of the desuccinylation of pyruvate dehydrogenase mediated by SIRT5 mitigates murine burn sepsis.^32^ Using a Co‐IP assay, we validated that SIRT5 overexpression reversed the LPS‐induced increase in HOXA5 succinylation. Therefore, we observed a decrease in SIRT5‐mediated HOXA5 desuccinylation in LPS‐stimulated MLE‐12 cells. Moreover, *HOXA5* depletion neutralized *SIRT5* overexpression‐induced inhibition of ferroptosis. Thus, *SIRT5* overexpression increased HOXA5 desuccinylation, thereby inhibiting ferroptosis in sepsis‐induced lung injury.

In summary, our findings confirmed that SIRT5 mediates HOXA5 desuccinylation, which activates *FSP1* transcription, inhibits ferroptosis, and alleviates LPS‐induced septic lung injury. This study provides insights for the identification of novel therapeutic targets for sepsis‐induced lung injury.

## CONFLICT OF INTEREST STATEMENT

The authors declare no conflicts of interest.

## Supporting information


**Figure S1.** GPX4 expression levels in MLE‐12 cells after corresponding treatments.

**Figure S2.** HOXA5 suppresses ferroptosis by binding to the GPX4 promoter in LPS‐induced septic lung injury in vitro.

## Data Availability

Data sharing is not applicable to this article as no datasets were generated or analyzed during the current study.
