# Supplementary figures and images for: SIRT5‐mediated HOXA5 desuccinylation inhibits ferroptosis to alleviate sepsis induced‐lung injury
Source: Kaohsiung J Med Sci. 2024 Dec 23;41(1):e12921. doi: 10.1002/kjm2.12921 (PMC11724168; doi:10.1002/kjm2.12921)

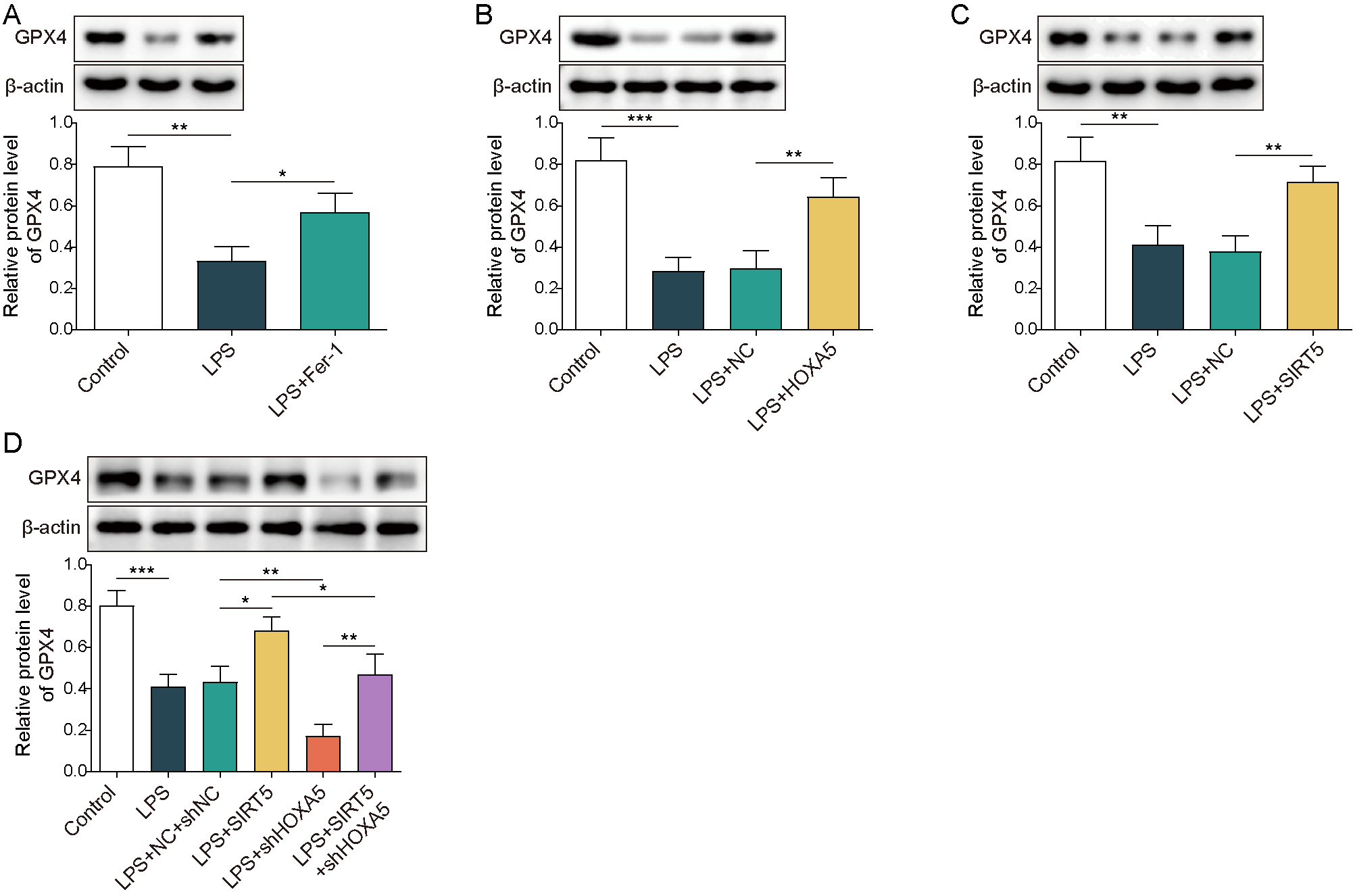

Supplement: Supplementary file 1 — Figure S1. GPX4 expression levels in MLE‐12 cells after corresponding treatments. [file KJM2-41-e12921-s001.tif]

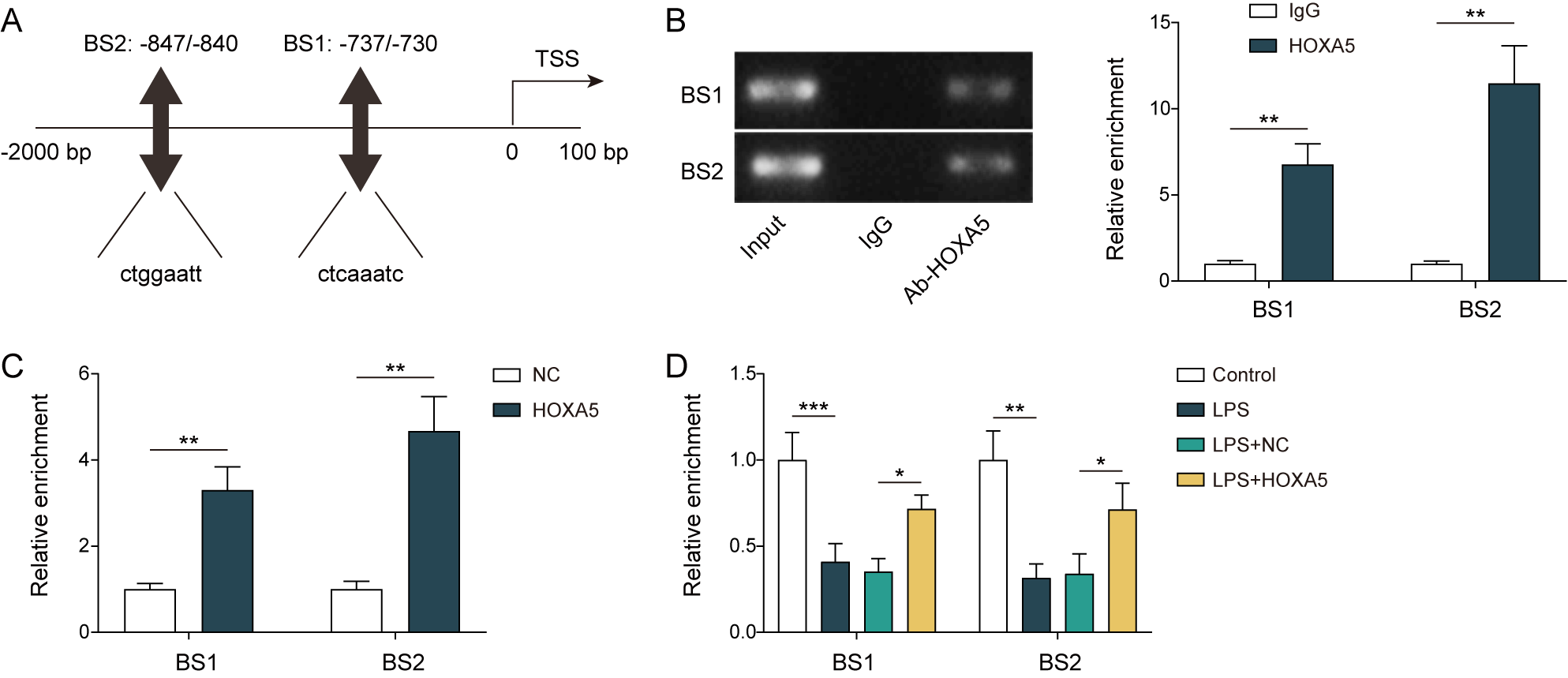

Supplement: Supplementary file 2 — Figure S2. HOXA5 suppresses ferroptosis by binding to the GPX4 promoter in LPS‐induced septic lung injury in vitro. [file KJM2-41-e12921-s002.tif]
